# Palliative radiotherapy of bone metastases in octogenarians: How do the oldest olds respond? Results from a tertiary cancer center with 288 treated patients

**DOI:** 10.1186/s13014-022-02122-2

**Published:** 2022-09-07

**Authors:** Alexander Rühle, Verlaine Ange Nya Yompang, Simon K. B. Spohn, Raluca Stoian, Constantinos Zamboglou, Eleni Gkika, Anca-Ligia Grosu, Nils H. Nicolay, Tanja Sprave

**Affiliations:** 1 Department of Radiation Oncology, University of Freiburg - Medical Center, Robert-Koch-Str. 3, 79106 Freiburg, Germany; 2 German Cancer Consortium (DKTK) Partner Site Freiburg, German Cancer Research Center (DKFZ), Heidelberg, Germany

**Keywords:** Radiotherapy, Elderly, Geriatric, Bone metastases, Octogenarians, SINS

## Abstract

**Background:**

Accompanied by the demographic change, the number of octogenarian cancer patients with bone metastases will increase in the future. Palliative radiotherapy constitutes an effective analgesic treatment; however, as pain perception and bone metabolism change with increasing age, the analgesic efficacy of radiotherapy may be altered in elderly patients. We therefore investigated the treatment outcomes of palliative radiotherapy for bone metastases in octogenarians.

**Methods:**

Patients between 80 and 89 years undergoing radiotherapy for bone metastases between 2009 and 2019 at a tertiary cancer center were analyzed for patterns-of-care, pain response and overall survival (OS). Logistic regression analyses were carried out to examine parameters associated with pain response, and Cox analyses were conducted to reveal prognostic parameters for OS.

**Results:**

A total of 288 patients with 516 irradiated lesions were included in the analysis. The majority (n = 249, 86%) completed all courses of radiotherapy. Radiotherapy led to pain reduction in 176 patients (61%) at the end of treatment. Complete pain relief at the first follow-up was achieved in 84 patients (29%). Bisphosphonate administration was significantly associated with higher rates of pain response at the first follow-up (*p* < 0.05). Median OS amounted to 9 months, and 1-year, 2-year and 3-year OS were 43%, 28% and 17%. In the multivariate analysis, ECOG (*p* < 0.001), Mizumoto score (*p* < 0.01) and Spinal Instability Neoplastic Score (SINS) (*p* < 0.001) were independent prognosticators for OS.

**Conclusion:**

Palliative radiotherapy for bone metastases constitutes a feasible and effective analgesic treatment in octogenarian patients. ECOG, Mizumoto score and SINS are prognosic variables for survival and may aid treatment decisions regarding radiotherapy fractionation in this patient group. Single-fraction radiotherapy with 8 Gy should be applied for patients with uncomplicated bone metastases and poor prognosis. Prospective trials focusing on quality of life of these very old cancer patients with bone metastases are warranted to reveal the optimal radiotherapeutic management for this vulnerable population.

**Supplementary Information:**

The online version contains supplementary material available at 10.1186/s13014-022-02122-2.

## Introduction

The proportion of elderly cancer patients is prognosticated to dramatically increase over the following decades [1]. In 2050, 6.9 million new cancers are estimated to be diagnosed in patients aged ≥ 80 years or older worldwide, which would represent 20.5% of all cancer cases [1]. Given the high frequency of bone metastases in metastasized cancer patients [2], the number of elderly cancer patients with bone metastases will rise accordingly. As this population is underrepresented in many clinical trials, treatment decision making is challenging [3].

Immobility and pain are frequent symptoms caused by bone metastases, significantly impairing patients’ quality of life (QoL) [4]. In general, patients with bone metastases benefit from optimum multidisciplinary management of a multidisciplinary team in which all available treatments such as palliative radiotherapy, orthopedic surgery, radionuclide therapy and systemic treatments including bone-modifying agents (i.e., bisphosphonates and denosumab) are discussed in light of different patient- and tumor-related parameters such as the imaging findings, primary cancer, overall prognosis, performance status, symptoms and patient preferences [5–7]. Due to the often-impaired general performance status, frailty and high comorbidity burden of very old cancer patients [8, 9], palliative radiotherapy of bone metastases is often preferred over surgical approaches in these patients [10]. Because of the high incidence of comorbidities such as diabetes, kidney failure and osteoporosis, all possibly affecting bone metabolism, in the elderly population, the likelihood for pathological fractures and pain response after palliative radiotherapy may be different between younger and elderly patients. For instance, pain perception has been shown to vary by age [11], raising the question whether palliative radiotherapy is an effective treatment in patients with very advanced age. Furthermore, very elderly patients with metastatic disease often do not receive systemic anti-cancer treatments, possibly affecting survival rates in these patients [12]. Treatment goals can vary between younger and elderly cancer patients with metastatic disease [13], for instance, elderly patients in general often rank maintaining independence as very important treatment goal [14]. Whereas more and more studies have demonstrated both the feasibility and the effectiveness regarding radiotherapy for (very) old cancer patients [15–19], palliative radiotherapy for bone metastases of octogenarians (80–89 years) is poorly studied in the literature.

Here, we present the outcome data for palliative radiotherapy of bone metastases in a large cohort of octogenarian cancer patients treated within a tertiary cancer center. We investigated the feasibility and analgesic efficacy of palliative radiotherapy, analyzed potential predictors for pain reduction, and identified risk factors for overall survival (OS).

## Material and methods

### Data collection, treatment, and follow-up consultation

All cancer patients aged 80–89 years who received palliative radiotherapy for bone metastases between 2009 and 2019 at the Department of Radiation Oncology, Medical Center – University of Freiburg, were eligible for this analysis. Demographic characteristics, treatment parameters and pain response were retrospectively extracted from the electronic patient records. For spinal metastases, both the Spinal Instability Neoplastic Score (SINS) and the Mizumoto score were retrospectively computed at baseline. For bone metastases of the long bones, the Mirels score was computed following the initial publication [20]. The scores were determined by a board-certified radiation oncologist. If patients exhibited more than one irradiated spinal metastasis, the worst Mizumoto and SINS value was used for further analyses.

In general, treatment decisions especially for potentially unstable bone metastases were based on multidisciplinary tumor board recommendations. Patients usually underwent three-dimensional conformal radiotherapy. Radiotherapy planning was performed with either Oncentra MasterPlan® (Nucletron BV, Veenendaal, the Netherlands) or Eclipse™ planning systems (Varian Medical Systems, Palo Alto, CA, USA). Radiotherapy treatment plans were reviewed by at least two board-certified radiation oncologists. Decisions regarding radiation dose, fractionation and target volume were based on patients’ performance status, overall prognosis, pain intensity, tumor histology and patient preferences. In case of upfront surgery, postoperative radiotherapy was applied after a minimum of about 2 weeks to ensure sufficient wound healing. For cervical spine metastases or bone metastases in the skull base or head-and-neck region, patients were immobilized with individually moulded thermoplastic masks. In order to ensure exact positioning of patients in case of bone metastases at patients’ extremities, an individually moulded cast was used in most cases. Patients were offered at least one follow-up consultation at 6 weeks after completion of radiotherapy in order to evaluate the treatment response. This follow-up was performed as face-to-face consultation in the Department of Radiation Oncology, Medical Center – University of Freiburg. Due to the retrospective nature of the study, neither Numerical Rating Scale (NRS) or Visual Analogue Scale (VAS) values could sufficiently be assessed in a significant number of patients, so that pain improvement, defined as patient-reported decrease in pain intensity or ≥ 25% reduction in opioid intake with at least stable pain intensity, and complete pain relief were extracted from the electronic patient charts. The Independent Ethics Committee of the Medical Faculty, University of Freiburg approved this analysis in advance (Record No. 376/19).

### Statistics

Depending on the variable, patient and treatment characteristics were presented as frequencies or median values including the interquartile range (IQR). OS was calculated from the start of radiotherapy until death. Patients were censored at the last date the patient was known to be alive, and missing survival data were acquired through the Comprehensive Cancer Center Freiburg. Kaplan–Meier survival curves for OS including the corresponding 95% confidence intervals (95% CI) were presented, and log-rank tests were used to compare OS curves. Cox proportional hazards regression analyses for OS were carried out, and hazard ratios (HR) with the corresponding 95% CI were presented. Parameters that exhibited a *p* value < 0.1 in the univariate Cox analysis were included in the multivariate analysis, in which all variables were entered in the model in one single step (enter method). A logistic regression analyses with pain response at the first follow-up as dependent variable was conducted to reveal potential parameters associated with pain response. Odds ratios (OR) with the corresponding 95% CI were indicated. *P* < 0.05 was considered statistically significant throughout the study. SPSS Statistics software version 25 (IBM, Armonk, NY, USA) was used for statistical analyses, while GraphPad version 8.2.1 (GraphPad Software, San Diego, CA, USA) was used for visualization of the Kaplan–Meier curves.

## Results

### Patient and treatment characteristics

A total of 288 patients with 516 irradiated target volumes met the inclusion criteria and were analyzed. Median age of the cohort amounted to 82 years, and about one third of the study population (n = 85, 29.5%) were between 85 and 89 years old (Table 1). There was a slight predominance of men within our cohort (n = 167, 58.0%), similar to previously reported studies [9, 21]. About half of patients exhibited an ECOG status of 1 (n = 141, 49.0%), whereas only 49 patients (17.0%) had an ECOG status of 0. The majority of patients underwent palliative radiotherapy as inpatient (n = 172, 59.7%). The most common primary malignancies were genitourinary cancers (n = 117, 40.6%) including prostate cancer (n = 91, 31.6%), followed by breast cancer (n = 67, 23.3%) and lung cancer (n = 40, 13.9%).

**Table 1:** Patient characteristics of 288 octogenarian cancer patients with bone metastases receiving radiotherapy between 2009 and 2019

	n	%
*Gender*		
Male	167	58.0
Female	121	42.0
*Age*		
80–84 years	203	70.5
85–89 years	85	29.5
*ECOG*		
0	49	17.0
1	141	49.0
2	91	31.6
3	6	2.1
4	1	0.3
*Primary cancer*		
Genitourinary	117	40.6
Breast	67	23.3
Lung	40	13.9
Gastrointestinal	18	6.3
Hepatocellular/pancreatobiliary	10	3.5
Gynecological^a^	9	3.1
Thyroid	3	1.0
Head-and-neck	2	0.7
Skin	2	0.7
Others^b^	3	1.0
Unknown	17	5.9
*Hospitalization*		
Inpatient	172	59.7
Outpatient	116	40.3

^a^Vulva, cervix, uterus, ovar

^b^Angiosarcoma, pleural mesothelioma, malignant nerve sheath tumor

A total of 249 patients (86.5%) completed all radiotherapy courses by receiving the radiotherapy dose that was initially prescribed (Table 2). Of the 516 treated lesions, 455 (83.9%) received the initially prescribed dose. In average, a median dose of 35 Gy (IQR 30–35 Gy) delivered in 12 fractions (IQR 10–14) was applied. Short-course regimens consisting of five fractions or less were performed for 52 lesions (10.1%). The most common fractionation schedules were 10 × 3 Gy (n = 148), 14 × 2.5 Gy (n = 136) and 12 × 3 Gy (n = 76), while 5 × 4 Gy was applied in 11 cases, and 1 × 8 Gy in 9 cases. The most common reasons for radiotherapy discontinuation (n = 39) were death during radiotherapy (n = 19, 48.7%) and worsening of patients’ general condition (n = 14, 35.9%). Seven patients (2.4%) received re-irradiation of a previously irradiated lesion (n = 9). The majority of the 516 target volumes were located in the spine (n=254, 49.2%) and in the pelvis (n=153, 29.7%). Other common localizations were the thorax (n = 37, 7.2%) and the lower extremities (n = 29, 5.6%). The SINS was calculated for spine metastases and ranged at 7 in median (IQR 5–10). Only 12 lesions (5.6%) exhibited a SINS of ≥ 13 and were therefore classified as unstable. About two third (n = 140, 64.8%) of patients with spine metastases exhibited a Mizumoto score of 0–4 (group A), whereas 74 patients (34.3%) had a score of 5–9 (group B). Only 2 patients (0.9%) in our cohort were found to belong to group C (10–14 points). The Mirels score of long bone metastases amounted to median 9 points (IQR 7–9). Among the 516 treated lesions, a total of 135 (26.2%) were considered as complicated.

**Table 2:** Treatment characteristics regarding palliative radiotherapy of spinal cord metastases of 288 octogenarian cancer patients

	n	%
*Radiotherapy completion*		
Completed	249	86.5
Discontinued	39	13.5
*Reason for discontinuation (n = 39)*		
Death during radiotherapy	19	48.7
Patient’s wish	5	12.8
Worsening of general condition	14	35.9
Unknown	1	2.6
*Localization (n = 516)*		
Head-and-neck (w/o cervical spine)	15	2.9
Spine	254	49.2
Pelvis	153	29.7
Thorax (w/o thoracic spine)	37	7.2
Upper extremity	28	5.4
Lower extremity	29	5.6
*SINS for spine metastases (n = 216)*^a^		
0–6 (stable)	102	47.2
7–12 (possibly impending)	102	47.2
13–18 (unstable)	12	5.6
*Mizumoto score for spine metastases (n = 216)*^a^		
0–4	140	64.8
5–9	74	34.3
10–14	2	0.9
*Orthopedic corset*		
No orthopedic corset	258	89.6
Orthopedic corset	30	10.4
*Bone-modifying agents*		
No bone-modifying agents	161	55.9
Bisphosphonates	105	36.5
Denosumab	20	6.9
Unknown	2	0.7
*Systemic anti-cancer treatment*		
No systemic treatment	209	72.6
Systemic treatment	77	26.7
Unknown	2	0.7

	Median	IQR
*Radiotherapy fractionation (n = 516)*		
Number of fractions	12	10–14
Single dose [Gy]	3	2.5–3
Total dose [Gy]	35	30–35

*IQR* interquartile range, *SINS* Spinal Instability Neoplastic Score, *w/o* without

^a^If patients exhibited more than one irradiated spinal metastasis, the worst Mizumoto and SINS value is presented

About one in ten patients (n = 30, 10.4%) was fitted with an orthopedic corset during the course of radiotherapy. The most commonly prescribed bone-modifying agents were bisphosphonates (n = 105, 36.5%) followed by denosumab (n = 20, 6.9%). One fourth of the cohort (n = 77, 26.7%) received systemic anti-cancer therapy including endocrine therapy.

### Pain response

Palliative radiotherapy resulted in pain reduction in 176 patients (61.1%) at the end of treatment, whereas 88 patients (30.6%) reported equal pain levels. Only a minority (n = 13, 4.5%) suffered from increased pain severity at the end of treatment. A complete pain relief at the first follow-up consultation was achieved in 84 patients (29.1% of all treated patients, 53.8% of 156 patients attending the first follow-up consultation), while pain improvement compared to baseline pain level was reported in a total of 143 cases (49.7% of all patients, 91.7% of patients attending the first follow-up appointment). Table 3 shows the logistic regression analysis with pain response at the first follow-up consultation being the dependent variable. Here, bisphosphonate administration was significantly associated with higher rates of pain response at the first follow-up (OR = 0.084, 95% CI 0.011–0.619, *p* = 0.015). All other analyzed parameters had no impact on the probability of pain response at the first follow-up assessment.

**Table 3:** Logistic regression in order to identify potential parameters associated with pain response at first follow-up consultation

Univariate analysis	OR	95% CI	*p* value
Age (continuous)	1.053	0.770–1.439	0.747
ECOG (continuous)	0.327	0.099–1.079	0.066
Gender (reference: female)	2.297	0.314–16.802	0.413
Systemic treatment (reference: systemic treatment)	2.652	0.565–12.445	0.216
Orthopedic corset (reference: orthopedic corset)	5.253	0.552–49.982	0.149
Bisphosphonates (reference: bisphosphonates)	0.084	0.011–0.619	0.015
Denosumab (reference: denosumab)	0.669	0.057–7.860	0.749
Primary cancer (reference: genitourinary)	2.524	0.354–18.002	0.356
Primary cancer (reference: breast)	1.509	0.137–16.577	0.737
SINS (continuous)	1.228	0.916–1.647	0.169
Mizumoto score (continuous)	1.057	0.678–1.649	0.805

*ECOG* Eastern Cooperative Oncology Group, *SINS* Spinal Instability Neoplastic Score

Odds Ratios (ORs) with the 95% confidence intervals (95% CI) and the corresponding *p* values are shown

### Treatment outcomes

**Fig. 1:**
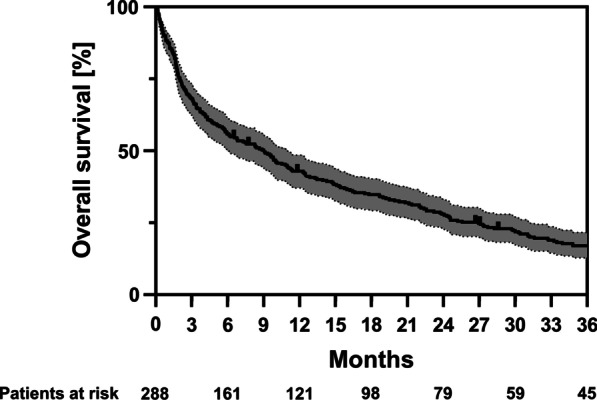
Kaplan–Meier curve for OS of octogenarians who received palliative radiotherapy for bone metastases between 2009 and 2019 (n = 288). The 95% confidence interval is displayed in grey

**Fig. 2:**
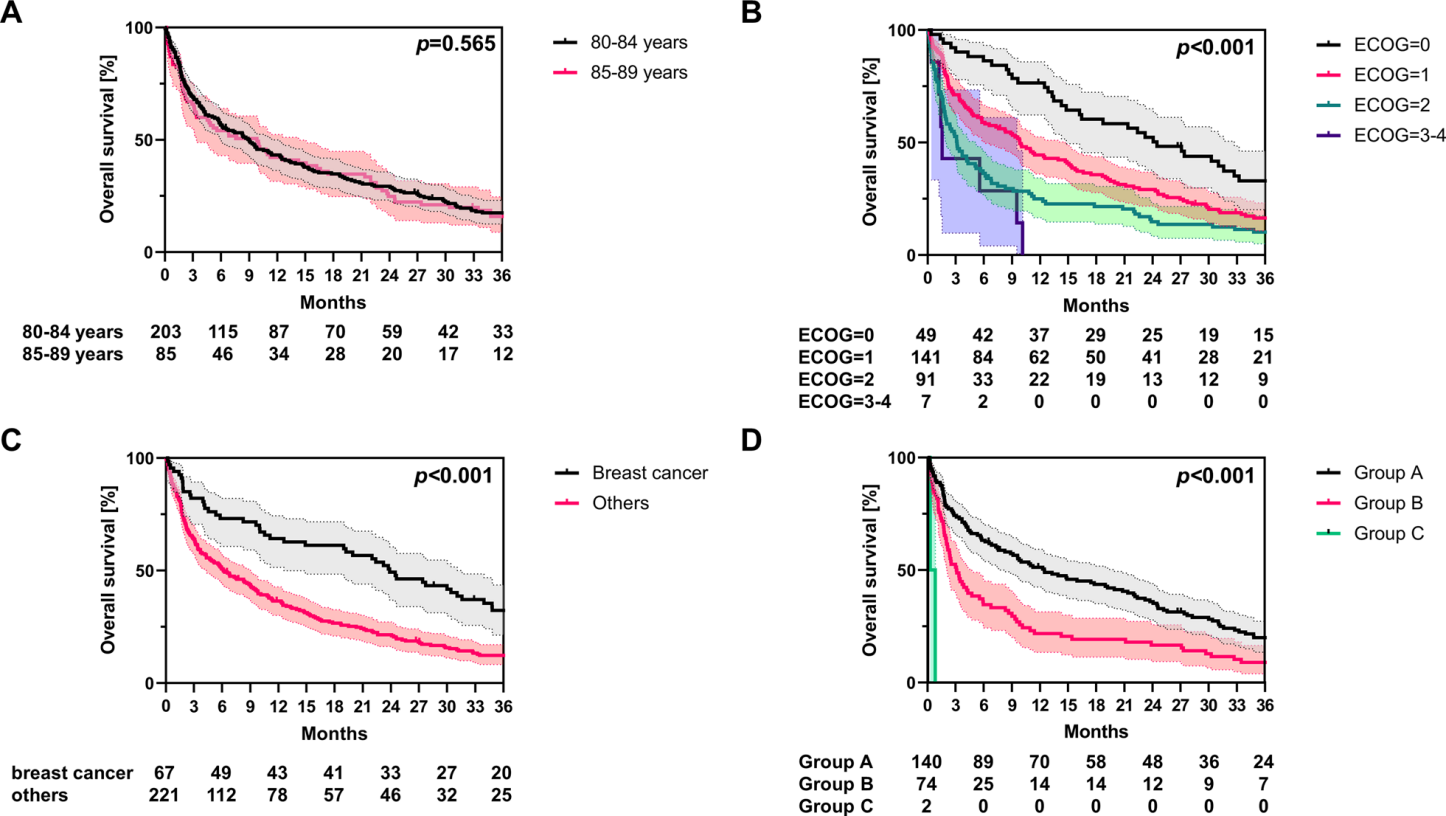
OS of octogenarians who received palliative radiotherapy for bone metastases between 2009 and 2019 (n = 288) depending on age (**A**), ECOG performance status (**B**), primary cancer (**C**), and Mizumoto score (n = 216 with irradiated spine metastases) (**D**). Log-rank tests were performed for comparisons of the different groups, and 95% confidence intervals are shown as pale colors

At the time of data analysis, 263 of the 288 patients had died. Median OS amounted to 9 months, and 1-year, 2-year and 3-year OS were 43%, 28% and 17%, respectively (Fig. 1). Cox proportional hazards regression analyses were conducted to reveal potential prognostic value of patient- and tumor-related parameters. Interestingly, age itself was not prognostic for OS in octogenarian cancer patients with bone metastases receiving palliative radiotherapy (HR = 1.017, 95% CI 0.973–1.063, *p* = 0.456) (Table 4). Figure 2A shows the Kaplan–Meier curves for patients aged 80–84 years versus patients aged 85 years or older (*p* = 0.565, log-rank test). In contrast, ECOG performance status was found to significantly stratify patients’ prognosis (Fig. 2B): Median OS ranged at 24 months, 10 months and 3 months for patients exhibiting an ECOG of 0, 1 or 2, respectively (HR = 1.641, 95% CI 1.398–1.927, *p* < 0.001), Median OS amounted to only 47 days, if the ECOG performance status was 3 or 4. Female gender was a further prognosticator for improved OS in the univariate but not in the multivariate analysis (Additional file 1: Fig. S1): Median OS was more than double as long for women (15 months) compared to men (7 months).

**Table 4:** Univariate and multivariate Cox analysis of several parameters regarding OS in octogenarians receiving palliative radiotherapy for bone metastases (n = 288)

	HR	95% CI	*p *value
*Univariate analysis*			
Age (continuous)	1.017	0.973–1.063	0.456
ECOG (continuous)	1.641	1.398–1.927	**< 0.001**
Gender (reference: female)	1.483	1.156–1.903	**0.002**
Systemic treatment (reference: systemic treatment)	0.887	0.676–1.165	0.390
Primary cancer (reference: genitourinary)	1.038	0.810–1.329	0.770
Primary cancer (reference: breast)	1.914	1.423–2.576	**< 0.001**
Mizumoto score (continuous)	1.164	1.080–1.255	**< 0.001**
SINS (continuous)	1.076	1.029–1.126	**0.001**
*Multivariate analysis*			
ECOG (continuous)	1.411	1.174–1.695	**< 0.001**
Gender (reference: female)	0.731	0.473–1.130	0.159
Primary cancer (reference: breast)	1.578	0.934–2.667	0.088
Mizumoto score (continuous)	1.127	1.040–1.222	**0.004**
SINS (continuous)	1.100	1.047–1.155	**< 0.001**

Significant p values (< 0.05) are expressed in bold

*ECOG* Eastern Cooperative Oncology Group, *SINS* Spinal Instability Neoplastic Score

Pre-therapeutic parameters which exhibited a *p* value < 0.1 in the univariate analysis were included in the multivariate analysis. In the multivariate analysis, all variables were entered in the model in one single step (enter method). Hazard ratios (HRs) and the corresponding 95% confidence intervals (95% CI) are shown

Absence of systemic anti-cancer treatment was not associated with inferior OS in our analysis (HR = 0.887, 95% CI 0.676–1.165, *p* = 0.390). Whereas patients with genitourinary cancers as primary did not exhibit better OS rates (HR = 0.964, 95% CI 0.752–1.235, *p* = 0.770), octogenarian patients with bone metastases deriving from breast cancer had significantly superior survival rates in our cohort (HR = 0.522, 95% CI 0.388–0.703, *p* < 0.001) (Fig. 2C). Higher SINS values, indicating a higher probability of potential instability, were associated with significantly diminished OS (HR = 1.076, 95% CI 1.029–1.126, *p* = 0.001). Increasing Mizumoto scores went along with significantly reduced OS rates (HR = 1.164, 95% CI 1.080–1.255, *p* < 0.001): Median OS was 12 months for group A (0–4 points) versus 3 months for group B (5–9 months) (*p* < 0.001, log-rank test). The two patients in group C (10–14 points) died after 9 and 25 days, respectively (Fig. 2D).

In the multivariate analysis, a higher ECOG performance status (HR = 1.411, 95% CI 1.174–1.695, *p* < 0.001), higher Mizumoto scores (HR = 1.127, 95% CI 1.040–1.222, *p* = 0.004) and increased SINS values (HR = 1.100, 95% CI 1.047–1.155, *p* < 0.001) remained independent prognosticators for reduced OS, whereas male gender (HR = 0.731, 95% CI 0.473–1.130, *p* = 0.159) did not. Non-breast cancer histology was found to be a borderline risk factor in the multivariate analysis (HR = 1.578, 95% CI 0.934–2.667, *p* = 0.088).

## Discussion

In this large single-center analysis regarding the outcomes of palliative radiotherapy for bone metastases of octogenarian patients, we could demonstrate that palliative radiotherapy was feasible and could be completed in about 85%. Palliative radiotherapy led to pain reduction in more than 60% of patients at the end of radiotherapy, and 29% of treated patients reported about absent pain at the first follow-up consultation. Only absent bisphosphonate administration was significantly linked to lower rates of pain response at the first follow-up. ECOG performance status but not age itself was found to serve as an independent prognosticator for OS in this very old population. Higher Mizumoto and SINS values were found to be further independent risk factors for reduced OS in octogenarian patients receiving palliative radiotherapy for bone metastases, while the prognostic value of non-breast cancer histology was of borderline significance in the multivariate analysis.

The strong prognostic value of patients’ performance status for OS is in line with previous analyses of elderly patients with bone metastases undergoing radiotherapy [22, 23]. Given the very poor outcomes of patients with an ECOG performance status of 2 or worse (median < 3 months), short-course radiotherapy should be preferred in these patients in order to avoid unnecessarily high treatment time spent in hospitals within the remaining life span. Randomized clinical trials and a meta-analysis have proven equivalence of single-fraction palliative radiotherapy regimens (single fraction of 8 Gy) with multi-fraction regimens (e.g., 10 fractions of 3 Gy) in terms of acute toxicities and pain response [24–32]. However, re-treatment rates were found to be considerably higher (20% versus 8%) after single-fraction radiotherapy in the updated meta-analysis of Rich and colleagues [32]. According to the current ASTRO guideline concerning palliative radiotherapy for bone metastases, single 8 Gy fraction is particularly convenient for patients with limited life expectancy [7]. A recent phase I/II trial of the SHARON project investigated short-course radiotherapy courses in which an accelerated hypofractionation radiotherapy regimen (2 days with twice daily fractionation) was delivered [33]. Such a scheme, e.g., 20 Gy in 5 Gy delivered twice daily, was found to result in favorable pain response (overall response: 84.0%, complete response: 32.0%) and low acute toxicity rates (grade ≥ 2 acute toxicities: 16.1%) in patients with complicated bone metastases. Such a concept can considerably reduce the overall time that patients need to stay in hospitals when compared to traditional regimens such as 30 Gy in 10 fractions, even though treatment time is still longer than single-fraction radiotherapy. However, further studies regarding these accelerated hypofractionation radiotherapy regimens are warranted.

The benefits of long-course radiotherapy courses, i.e., the reduced risk for re-irradiation [32], signs of higher re-mineralization rates in distinct patient populations (e.g., osteolytic metastases of breast cancer patients [34, 35]) and postponement/reduction of femoral fractures in femoral bone metastases [36, 37], must be critically weighted against the longer treatment time (in particular relevant considering the proportion of octogenarian patients treated as inpatient) and higher treatment costs [38]. Importantly, single-fraction radiotherapy with 8 Gy results in similar overall response rates compared to multi-fraction radiotherapy (72% vs. 75% among assessable patients in the updated meta-analysis of Rich et al. [32]) and is therefore especially convenient for patients with limited life expectance [7, 32]. According to the current ESTRO ACROP guideline, single-fraction radiotherapy with 8 Gy is the preferred radiotherapy schedule for uncomplicated bone metastases [39]. The re-irradiation rate in our study was relatively low (2.4% of treated patients) which may be related to the high proportion of patients receiving long-course radiotherapy, although other factors related to this special patient population (e.g., refusal of re-irradiation due to reduced performance status or impaired patient mobility) may also have contributed to this result, so that definitive conclusions based on our analysis are not possible.

In light of the high compliance rate and the relatively long survival time of patients with an ECOG status of 0 (median OS = 24 months) in our octogenarian cohort, long-course radiotherapy courses with focal dose escalation using simultaneous integrated boost concepts may increase the chance of long-term local control in these patients [40–43]. However, radiotherapy schedules with simultaneous integrated boosts are no standard treatments according to the current guidelines and require further prospective evidence [39, 44].

In the context of decision making for palliative radiotherapy of bone metastases, survival scores may be particularly helpful to decide between short-course and long-course palliative regimens [45]. Interestingly, although our patient cohort (median 82 years) was considerably older than the original publications of the Mizumoto score (median 63 years [46]), the Mizumoto score provided prognostic value even in the multivariate analysis. To the best of our knowledge, this is the first analysis showing a prognostic role of the Mizumoto score in this distinct group of very old cancer patients. We also could identify the SINS as independent prognosticator for OS, even though the scientific literature regarding the prognostic role of the SINS for OS is heterogeneous and most studied could not link the SINS to survival [47–50]. As the direct cause of death was not assessable in our study, we cannot prove whether there was a causal link also to disease-free survival. As higher SINS scores could more often lead to pain persistence (although not found in our analysis), immobility, secondary fractures and vertebral compression (parameters that could all potentially affect survival), it is not completely unreasonable to suspect a causal association [49, 51, 52].

The favorable survival rates of breast cancer patients in our study are in agreement with previous studies, e.g., the study of Ignat et al. or Bostel et al. [9, 53]. However, another study failed to show a prognostic role of breast cancer histology for patient survival [54]. The fact that the female gender was prognostic in the univariate but not in the multivariate Cox analysis is potentially related to its confounding role, as breast cancer histology tended to be associated with improved survival in the multivariate analysis. By combining the two favorable parameters ECOG = 0 and breast cancer histology, even octogenarian cancer patients with metastatic disease undergoing palliative radiotherapy exhibited relatively fair survival rates (median OS = 41 months), making other treatment goals besides pain control, i.e., bone stabilization, recalcification, and avoiding re-irradiation, more important for this subgroup. As recent analyses have shown promising results for stereotactic body radiotherapy (SBRT) of bone metastases in terms of long-term pain response and local control, this subgroup of patients may be appropriate for those approaches [55–58]. Considering the reduced time spent in hospitals in case of SBRT compared to long-course radiotherapy, this may especially be relevant for elderly patients whose general condition and mental state exhibit a higher possibility to decline after long-term hospital stays compared to their younger counterparts [59].

The favorable pain response rate in our octogenarian cancer patient cohort is in line with the Dutch Bone Metastasis Study which could show that age was not a predictor for pain response and that very old patients (≥ 75 years) had comparable outcomes in terms of pain response as younger patients (< 65 years) [60]. Importantly, elderly patients receiving palliative radiotherapy for bone metastases did exhibit similar overall QoL values as their younger counterparts [60]. Prescription of bisphosphonates was the only variable that was associated with higher pain response rates in our analysis. According to Cochrane systematic reviews, bisphosphonates have been shown to reduce both the incidence of skeletal‐related events and pain intensity in breast cancer patients with bone metastases [61], whereas there was no effect on pain response in prostate cancer patients [62, 63]. The ASCO recommends the usage of bone-modifying agents in metastatic breast cancer patients [64], and recommends consideration of bisphosphonate administration for castration-resistant prostate cancer patients with painful bone metastases [65]. The systematic review regarding the combination of bone-modifying agents and palliative radiotherapy for bone metastases concluded that there is insufficient evidence concerning a synergistic effect between both modalities [66]; however, at least animal studies showed that the addition of bisphosphonates to radiotherapy restored bone quality of metastatic lesions [67, 68]. Foerster et al. observed a trend (*p* = 0.069) towards higher increase in bone density of osteolytic bone metastases in breast cancer patients at 3 months after radiotherapy when radiotherapy was combined with bisphosphonates [69].

Surprisingly, when comparing our survival rates with other trials in which patients with irradiated bone metastases were included irrespectively of their age, the oncological outcomes of our study (1-year OS = 43%, 2-year OS = 28%) were found comparable. For instance, Katagiri et al. reported 1-year and 2-year OS rates of 36% and 23%, respectively, while it was 32% and 19% in the study of Mizumoto et al., respectively [46]. However, the median age in these studies was considerably lower (Katagiri et al.: 64 years, Mizumoto et al.: 63 years, therefore in average not considered as elderly) than in our study (82 years). These findings in combination with the absent prognostic role of age within our octogenarian cancer cohort underlines that the chronological age itself has limited prognostic value compared to other patient parameters in cancer patients with bone metastases [70]. In this context, it should not be forgotten that our cohort of octogenarian cancer patients that were referred to a tertiary cancer center for palliative radiotherapy of bone metastases may compromise a preselected group of rather healthier patients, as patients with a more impaired performance status may rather have received best supportive care in the first place.

Although our results are derived from a large cohort treated at a tertiary cancer center, several limitations should be noted. First, the typical flaws of retrospective analyses such as selection bias, observer bias and reporting bias are valid for our study, too. Systemic opioid and non-opioid analgesic treatments may have contributed to the relatively good pain response rates. Furthermore, as a decrease in opioid use is one criterion for pain response after palliative radiotherapy [71], patients with stable pain intensity but unknown reduction in opioid use may mistakenly been classified as non-responders. Administration of corticosteroids which was applied in patients with pain flare after start of radiotherapy may also have positively impacted patients’ pain intensity, especially regarding pain assessment at the end of the radiotherapy course. Second, we did not routinely perform routine CT-based re-staging during the follow-up in this vulnerable cohort so that comprehensive analyses about vertebral compression fractures and stabilization rates were not possible in our study. In another retrospective study of elderly cancer patients with spinal metastases treated by radiotherapy, recalcification and stabilization were evident in 40% of surviving patients after six months [9]. Third, both treatment-related toxicities and patient-reported outcomes were not assessed due to the retrospective nature of our study, making definitive conclusions regarding the therapeutic value of palliative radiotherapy for bone metastases in octogenarian cancer difficult.

However, our analysis provides promising results for palliative radiotherapy of bone metastases in octogenarian cancer patients in terms of patient compliance and pain response. The identified risk parameters for impaired OS (reduced performance status, higher Mizumoto and SINS values) may be used to stratify patients between short-course and long-course radiotherapy. Especially for uncomplicated bone metastases in patients with poor prognosis, single-fraction radiotherapy with 8 Gy should be applied. Prospective (randomized) trials comprising of these very old cancer with bone metastases are necessary to increase the scientific evidence on the ideal management for these special patient population [71]. Ideally, these studies should include QoL and other patient-reported outcomes as endpoints [72].

## Supplementary Information

**Additional file 1.** Overall survival of octogenarians undergoing palliative radiotherapy for bone metastases between 2009 and 2019 (n=288) in dependence of patients' gender.

## Data Availability

De-identified datasets used and analyzed during the current study are available from the corresponding author on reasonable request.
