# Supplementary figures and images for: Palliative radiotherapy of bone metastases in octogenarians: How do the oldest olds respond? Results from a tertiary cancer center with 288 treated patients
Source: Radiat Oncol. 2022 Sep 7;17:153. doi: 10.1186/s13014-022-02122-2 (PMC9450461; doi:10.1186/s13014-022-02122-2)

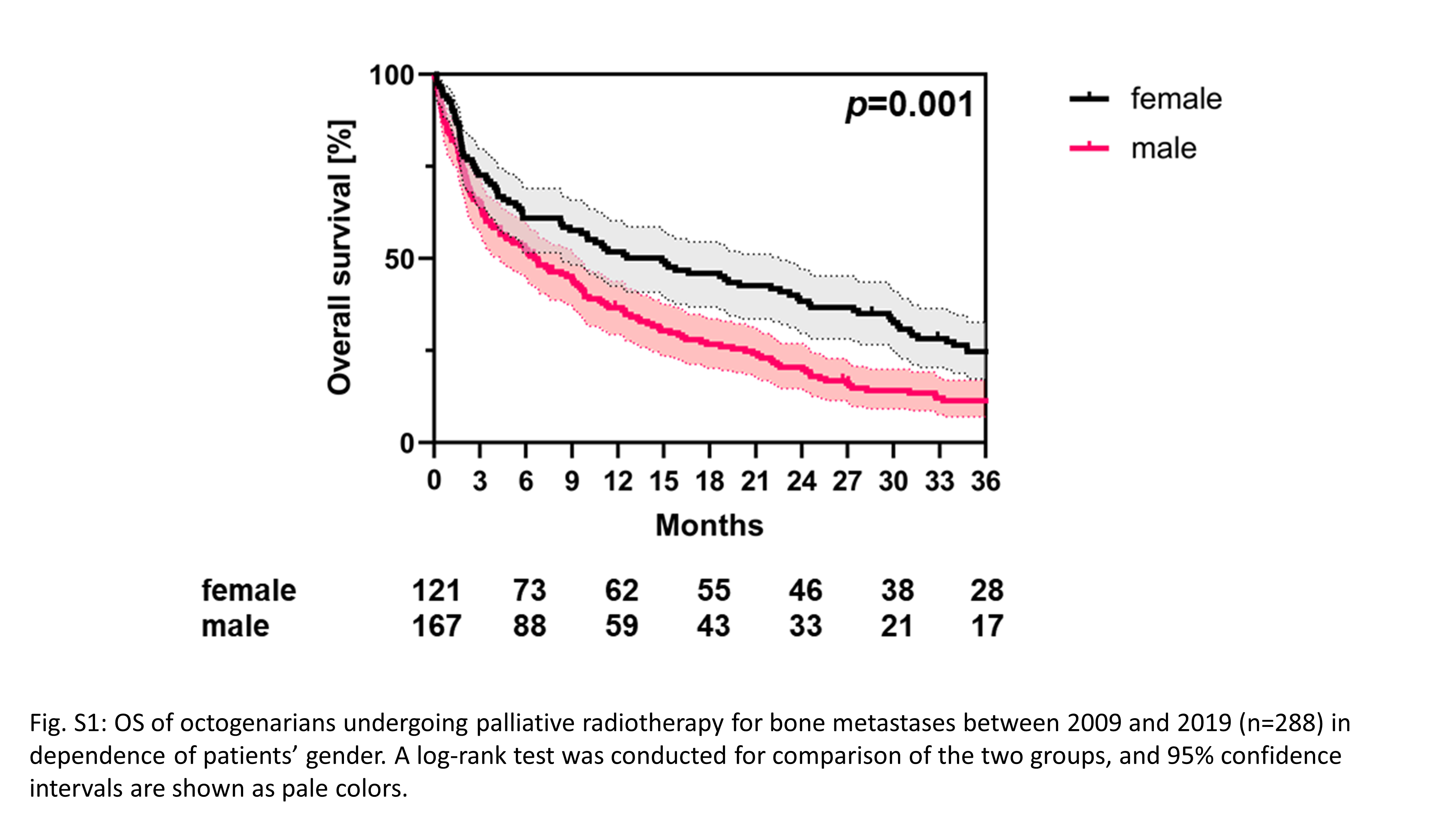

Supplement: Supplementary file 1 — Additional file 1. Overall survival of octogenarians undergoing palliative radiotherapy for bone metastases between 2009 and 2019 (n=288) in dependence of patients' gender. [file 13014_2022_2122_MOESM1_ESM.tif]
